# The Prognostic Value of Eight Immunohistochemical Markers Expressed in the Tumor Microenvironment and on Hodgkin Reed-Sternberg Cells in Pediatric Patients With Classical Hodgkin Lymphoma

**DOI:** 10.3389/pore.2022.1610482

**Published:** 2022-08-11

**Authors:** Eline A. M. Zijtregtop, Ilse Tromp, Rana Dandis, Christian M. Zwaan, King H. Lam, Friederike A. G. Meyer-Wentrup, Auke Beishuizen

**Affiliations:** ^1^ Department of Hemato-Oncology, Princess Máxima Center for Pediatric Oncology, Utrecht, Netherlands; ^2^ Department of Pediatric Hematology and Oncology, Erasmus Medical Center, Sophia Children’s Hospital, Rotterdam, Netherlands; ^3^ Department of Pathology, Erasmus Medical Center, Rotterdam, Netherlands

**Keywords:** prognosis, prognostic value, children, pediatric, classical Hodgkin lymphoma, immunohistochemical markers, prognostic markers

## Abstract

Immunohistochemical markers are associated with treatment outcome in adults with classical Hodgkin Lymphoma (cHL). Studies in children are scarce and inconsistent. We investigated in 67 children with cHL, whether the expression of CD15, CD30, PAX5, PD-1, PD-L1, CD68, CD163 and TARC at diagnosis is associated with disease free survival (DFS) and with interim remission status. Low CD15 and low TARC expression were associated with relapsed disease. Low expression of PD-L1 was associated with complete remission at interim PET-scan. Our data suggest a difference between pediatric and adult cHL. This underlines the importance of future research into specific prognostic factors in pediatric cHL, indispensable for improvement of treatment in this population.

## Introduction

Classical Hodgkin lymphoma (cHL) contains a notably small amount of 0.1%–10% malignant Hodgkin and Reed-Sternberg (HRS) cells, surrounded by benign inflammatory cells. These cells produce different cytokines and chemokines, maintaining a specific tumor microenvironment (TME) in which the HRS cells can thrive (1, 2). There is a strong variation among individual patients in the frequency and distribution of HRS cells and the cells of the TME (3). These variations may be used to identify prognostic markers in pediatric patients to develop risk-adapted treatment strategies, leading to better outcome and less treatment-related toxicities (1, 2). Furthermore, these markers could be new treatment targets. Multiple studies have demonstrated that the presence of certain immune cell types and immunohistochemical markers in the TME and on HRS cells is associated with treatment outcome (4–13). However, most studies have been performed in adult patients, and studies based on pediatric populations are scarce and inconsistent (8–10, 12). Previous studies have shown differences in TME composition, PD-L1 expression, and the role of Epstein Barr Virus (EBV) between pediatric and adult cHL patients (12, 14–17). So, it is uncertain whether outcomes of studies in adults with cHL are applicable to children with cHL.

Therefore, we investigated the prognostic value of eight different immunohistochemical markers in pediatric cHL. PD-1, PD-L1, CD68 and CD163 have previously been analyzed in pediatric studies and show conflicting outcomes (8–10, 12). TARC was chosen based on the prognostic impact in blood samples in both adult and pediatric studies (11, 18, 19). CD15, CD30 and PAX5 were chosen to confirm diagnosis of cHL, and the level of expression of these markers was analyzed as prognostic marker since there are some studies which found an association of this expression with survival (6, 9, 10).

## Methods

### Study Design and Participants

We performed this retrospective, explorative study at the Erasmus Medical Center—Sophia Children’s Hospital (Erasmus MC—Sophia) in Rotterdam, the Netherlands.

We collected 73 samples of children diagnosed with classical Hodgkin lymphoma (cHL) between 2000 and 2018. Inclusion criteria were 1) children up to the age of 18 years, 2) proven diagnosis of primary cHL. Exclusion criteria were 1) nodular lymphocyte-predominant Hodgkin lymphoma (NLPHL), 2) no representative lymphoid tissue available, 3) missing data regarding treatment outcome. An event-enriched cohort was created to increase the statistical power for our observations linked to the treatment outcome. To this end, we included all available patients with relapsed disease during the study period (*n* = 23). Afterwards, fifty patients who did not experience relapse were randomly selected and included (Supplementary Figure S1). In total, seventy-three patients were included. The informed consent procedure was conducted according to national guidelines.

### Data Collection

The study data were obtained from the patient database of the Erasmus MC—Sophia and the patient database of the Princess Máxima Center for Paediatric Oncology.

The following baseline characteristics were collected: age, sex, histologic subtype, staging conform the Ann Arbor staging system, and treatment protocol.

### Study Procedures

Potential predictors for the outcome were identified through an extensive literature search in PubMed (4–13, 17–19). We identified the following potential immunohistochemical markers: PD-1, PD-L1, CD15, CD30, CD68 CD163, TARC and PAX5.

Immunohistochemistry was performed with an automated, validated and accredited staining system (Ventana Benchmark ULTRA, Ventana Medical Systems, Tucson, AZ, United States) using ultra view or optiview universal DAB detection Kit. In brief, following deparaffinization and heat-induced antigen retrieval the tissue samples were incubated according to their optimized time with the antibody of interest (Supplementary Table S1). Incubation was followed by haematoxylin II counterstain for 12 min and then a blue colouring reagent for 8 min according to the manufactures instructions (Ventana).

**FIGURE 1:**
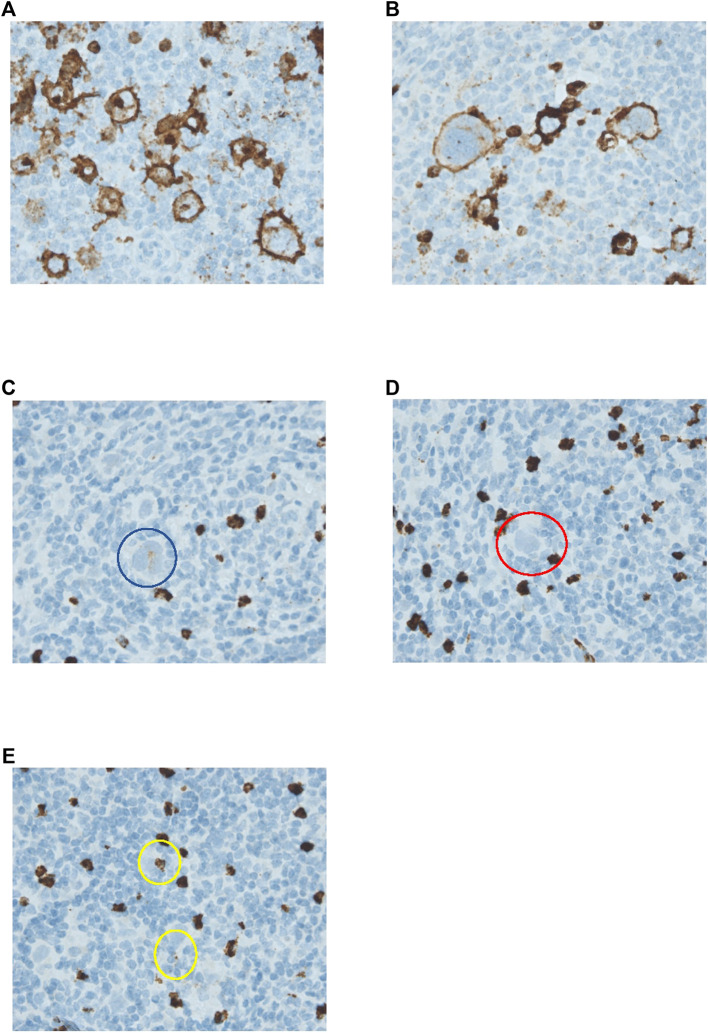
Immunohistochemical analysis of CD15 expression by HRS cells. **(A–E)** HRS cells stained for CD15. **(A)** HRS cells show both membranous and Golgi staining. **(B)** HRS cells show only membranous staining. Both **(A,B)** were counted as positive. **(C)** HRS cell circles in blue with weak positive staining. This cell was counted as positive. **(D)** HRS cell in red with negative staining. This cell was counted as negative. **(E)** Both of the staining patterns circled in yellow were considered to most likely be artefacts. These HRS cells were both counted as negative. All pictures were taken with a ×40 objective.

**FIGURE 2:**
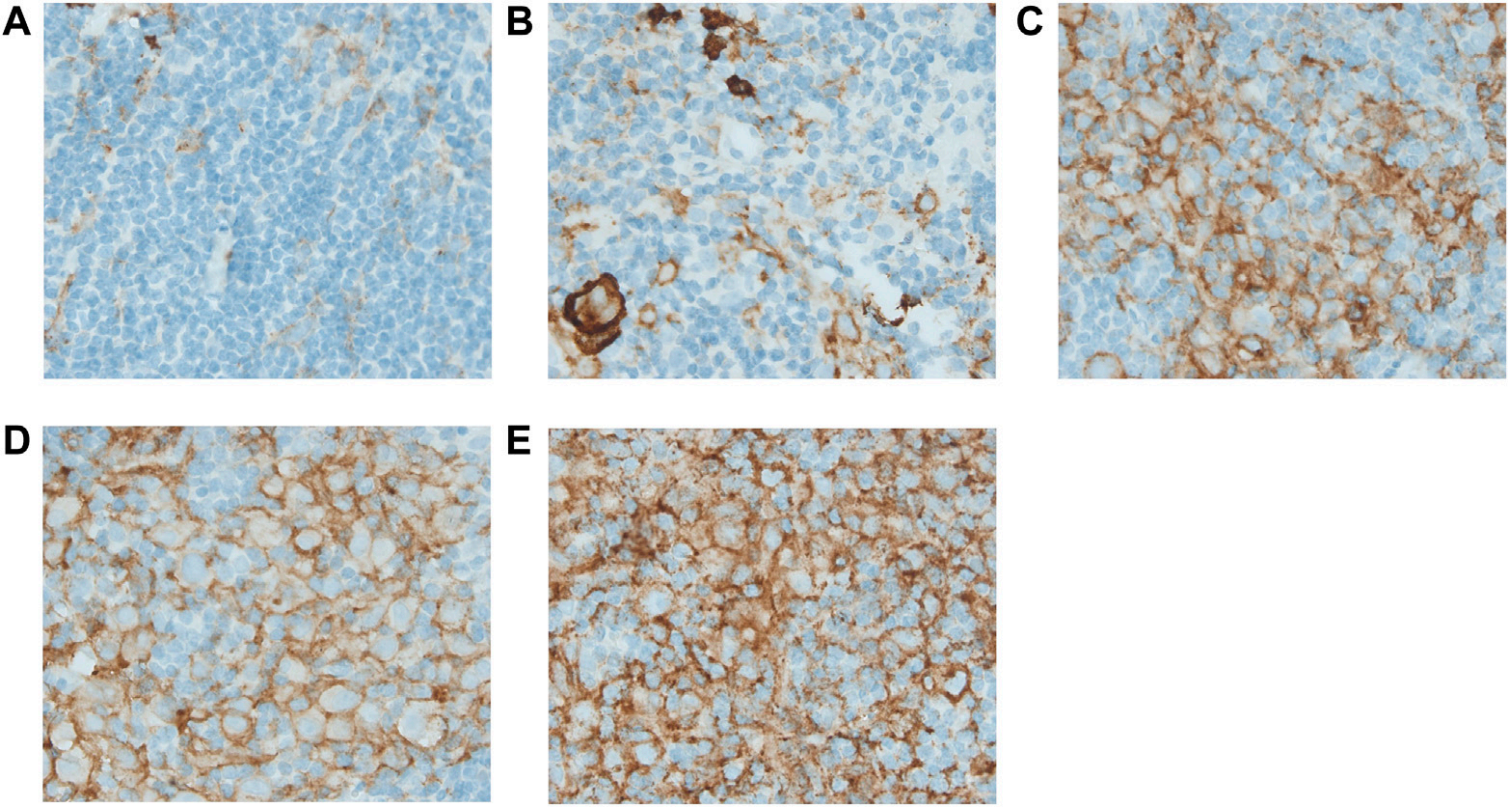
Immunohistochemical analysis of PD-L1 expressed in the tumour microenvironment. Figure 2 shows the estimates of the positive staining cells for PD-L1 in relation to the overall cellularity according to the five groups: 0–5% score 0 **(A)**, 6–25% score 1 **(B)**, 26–50% score 2 **(C)**, 51–75% score 3 **(D)**, and >75% score 4 **(E)**. All pictures were taken under a ×40 objective.

Scoring methods were based on the literature (4, 7, 13, 17). Tissue samples were reviewed by two individual researchers (K.L. and E.Z./I.T.), blinded for outcome. Expression for markers on HRS cells (CD15, CD30, PAX5, PD-L1, and TARC) was determined by counting 50 distinct HRS cells in representative high-power fields (Figure 1 and Supplementary Figures S2–S5) (17). Only the cells in which the morphological characteristics of HRS cells were clearly recognizable were scored. An extra scan was preformed after a field was scored to make sure no negative cells were missed. PD-L1 expression was scored positive if there was a membranous staining pattern. HRS cells that only had Golgi staining for PD-L1 were counted as negative. For markers in the TME (CD68, CD163, PD-1, and PD-L1) five neighbouring and representative high-power fields were observed, and estimates of the percentages of positive staining cells in relation to the overall cellularity were made according to five groups: 0–5% score 0, 6–25% score 1, 26–50% score 2, 51–75% score 3, and >75% score 4 (Figure 2 and Supplementary Figures S6–S8) (4, 7, 13).

### Study Outcomes

The primary endpoint of this study was treatment outcome, defined as disease free survival (DFS). DFS was defined as the absence of cHL progression or relapse. Treatment failure was defined as progression or relapse of the disease at any time during the study period. First, we investigated the association between the immunohistochemical markers and DFS. Secondly, we investigated the association between immunohistochemical marker expression and early and late relapse. Early relapse was defined as relapse <1 year after finishing treatment and late relapse as relapse >1 year after finishing treatment. The secondary endpoint of this study was the achievement of complete remission at the interim PET scan. In the non-European Network-Paediatric Hodgkin Lymphoma-C1/C2 (EuroNet-PHL-C1/C2) treatment protocols, instead of a PET scan, ultrasound evaluation or a computed tomography (CT) scan was used for interim evaluation (20, 21). A sub-analysis was done for the secondary outcome to try to reduce the chance of bias, only including patients treated according to the current EuroNet-PHL-C1/C2 protocols.

### Data Analysis

In order to study the univariate prognostic value of potential predictors, we applied the rule of thumb to reassure that we included at least 10 cases per variable. To examine the differences in patients’ characteristics at baseline the Pearson Chi-Square test, the Fisher’s exact test, the Independent Student’s t-test, and the Mann-Whitney test were used. For the primary outcome analysis, we performed the Mann-Whitney to investigate if there were differences in expression patterns between the different outcome groups. Afterwards, we performed receiver operating characteristics (ROC) analysis to check the discriminating power for DFS of each biomarker. For the secondary outcome analysis, we also investigated if there was an association between the different markers by Mann-Whitney test and we performed ROC curves to investigate the discriminative power for early versus late relapse or remission status. For remission status, a sub-analysis was performed for patients treated according to the EuroNet-PHL-C1/C2 treatment protocols. A 2-tailed *p*-value of <0.05 in the univariate analysis was considered statistically significant. IBM SPSS Statistics for Windows, Version 25.0 (IBM Corp., Armonk, NY, United States) and R software (R Foundation for Statistical Computing, Vienna, Austria) were used for the statistical analysis.

## Results

### Patient Inclusion and Baseline Characteristics

Seventy-three patients were included in the study. Six patients were excluded, based on the exclusion criteria (Supplementary Figure S1). The mean follow-up time was 5.4 years (range 1.6–7.2 years). Baseline characteristics for both the primary and secondary outcome groups are presented in Supplementary Table S2. Twenty-two out of the 67 patients (32.8%) experienced treatment failure, five of these patients (7.5%) passed away (Supplementary Figure S1). Of the patients with relapsed disease, ten patients (45%) had an early relapse of the disease within 1 year after treatment, and 12 patients (55%) had a late relapse. There were no differences in age, sex, histologic subtype and staging between the patients with treatment success and treatment failure. Forty-five patients (67.2%) were treated according to the EuroNet-PHL-C1/C2 protocols (22, 23).

Thirty patients (44.8%) did not achieve complete remission (CR) at interim PET scan. Significantly more females than males achieved CR (*p* = 0.012). Patients with lower stage were more likely to achieve CR at interim PET-scan (*p* = 0.033).

For both primary outcome and secondary outcome, there were significantly more patients with treatment success treated according to the EuroNet-PHL-C1/C2 treatment protocols compared to the other treatment protocols, and treated without radiotherapy versus with radiotherapy.

### Association Between Expression of Immunohistochemical Markers and Treatment Outcome

The Mann-Whitney test showed that the percentage of CD15 and TARC expression on HRS cells were associated with DFS. Patients with treatment failure had a significantly lower percentage of CD15 expression on HRS cells (p 0.002) and a significantly lower percentage of TARC expression on HRS cells (*p* = 0.019) (Table 1). For the other markers, there was no significant association with DFS (Table 1). After this analysis, we performed a ROC analysis to check the discriminative power of each marker. The percentage of CD15 expression showed adequate discriminative power for DFS with an AUC of 0.74 (95% CI 0.62–0.86) (Supplementary Figure S9). The percentage of TARC expression showed lower discriminative power for DFS with an AUC of 0.68 (95% CI 0.54–0.81) (Supplementary Figure S9). PD-L1 expression on HRS cells and PAX5 expression showed low, but significant, discriminative power for DFS with and AUC of 0.64 (95% CI 0.50–0.78) for PD-L1 and an AUC of 0.64 (95% CI 0.50–0.77) for PAX5. The other markers showed no significant discriminative power for DFS.

**TABLE 1:** Expression patterns of the different biomarkers regarding patients with treatment success versus patients with treatment failure.

	Treatment Success		Treatment Failure		*p* Value
	Value	n	Value	n	
Markers expressed on HRS cells (% positive cells)					
PAX5 Median (IQR)	90.00 (65.00–94.00)	45	79.00 (61.00–84.50)	22	0.071
CD15 Median (IQR)	86.00 (53.50–96.00)	44	51.00 (31.50–81.50)	22	0.002
CD30 Median (IQR)	94.00 (88.00–97.00)	45	95.00 (87.50–96.00)	22	0.552
PD-L1 Median (IQR)	96.00 (93.00–100.00)	45	96.00 (83.00–98.00)	22	0.056
TARC Median (IQR)	92.00 (88.00–96.00)	45	88.00 (77.50–92.00)	22	0.019
Markers in TME (score 0–4)					
CD163 Mean (SD)	1.99 (0.97)	45	2.41 (1.05)	22	0.134
CD68 Mean (SD)	2.45 (0.64)	45	2.37 (0.87)	22	0.394
PD-1 Mean (SD)	1.55 (0.94)	45	1.21 (0.98)	22	0.236
PD-L1 Mean (SD)	2.68 (0.73)	45	2.30 (0.95)	22	0.149

Abbreviations: HRS, Hodgkin and Reed-Sternberg; TME, tumour microenvironment; PAX5 Paired Box 5; PD-L1, programmed death ligand 1; TARC, thymus and activation-regulated chemokine; PD-1, programmed death 1.

There was not enough power to perform a sub-analysis with only the patients treated according to the EuroNet-PHL-C1/C2 treatment protocols. The sub-analysis to investigate the association between the immunohistochemical markers and early versus late relapse showed no association or discriminative power for any of the markers (Supplementary Table S3).

### Association Between Expression of Immunohistochemical Markers and Interim Positron Emission Tomography Scan

For the total group, there were no statistically significant differences in achievement of CR at the interim PET scan for expression of any of the markers (Supplementary Table S4). Notably, a sub-analysis, only including patients treated according to the EuroNet-PHL-C1/C2 protocols (*n* = 45), showed that expression of PD-L1 in the TME was associated with remission status. The Mann-Whitney test showed that patients with complete remission on interim PET-scan had significantly lower PD-L1 expression on the TME than patients with no complete remission at interim PET (*p* = 0.04) (Table 2). The ROC curve showed that PD-L1 had also discriminative power as marker for remission status as well, with an AUC of 0.69 (95% CI 0.53–0.85) (Supplementary Figure S10). The other markers showed no association or discriminative power in this analysis.

**TABLE 2:** Expression patterns of the different immunohistochemical markers regarding achievement of complete remission at interim PET scan for patients treated according to the EuroNet-PHL-C1/C2 protocols.

	Complete Remission at Interim PET Scan		No Complete Remission at Interim PET Scan		*p* Value
	Value	n	Value	n	
Immunohistochemical markers expressed on HRS cells (% positive cells)					
PAX5 Median (IQR)	91.00 (72.00–94.00)	30	90.00 (66.00–94.00)	15	0.923
CD15 Median (IQR)	88.00 (48.00–96.00)	29	64.00 (50.00–94.00)	15	0.434
CD30 Median (IQR)	95.00 (91.00–98.00)	30	96.00 (94.00–98.00)	15	0.650
PD-L1 Median (IQR)	97.00 (92.00–100.00)	30	98.00 (94.00–98.00)	15	0.941
TARC Median (IQR)	91.00 (88.00–96.00)	30	92.00 (82.00–96.00)	15	0.856
Immunohistochemical markers in TME (score 0–4)					
CD163 Mean (SD)	2.05 (1.02)	30	1.95 (1.01)	15	0.809
CD68 Mean (SD)	2.43 (0.65)	30	2.60 (0.71)	15	0.561
PD-1 Mean (SD)	1.41 (0.85)	30	1.80 (1.17)	15	0.201
PD-L1 Mean (SD)	2.49 (0.69)	30	3.00 (0.68)	15	0.040

Abbreviations: PET, positron emission tomography; HRS, Hodgkin and Reed-Sternberg; TME, tumour microenvironment; PD-L1, programmed death ligand 1; TARC, thymus and activation-regulated chemokine; PD-1, programmed death 1; IQR, interquartile range; SD, standard deviation.

## Discussion

The results of our study add significantly to the limited data available for pediatric cHL patients (8–10, 12). This is due to the high percentage of relapsed patients in our cohort and to the high number of markers analyzed.

We found that low CD15 expression on HRS cells at diagnosis was associated with relapsed disease. Previous studies described that loss of CD15 expression at diagnosis was associated with overall survival (OS) and failure-free survival (FFS) in pediatric patients and in adult patients (9, 24, 25). CD15 is expressed on HRS cells, but the exact role of CD15 is unclear. Our study is the first study that investigated the percentage of CD15 expression, and our data suggest that this percentage of CD15 expression is a prognostic factor for DFS in pediatric cHL.

We found that low expression of TARC on HRS cells was associated with treatment failure, although the discriminative power of TARC was limited. The impact of TARC expression on HRS cells has not been described in the literature yet. However, it is known that TARC plays a crucial role in the pathogenesis of cHL (19). The secretion of TARC by HRS cells causes a consequent attraction and homing of T-helper 2 (Th2) cells to the TME. These attracted Th2 cells can secrete Interleukin (IL)-4, IL-5 and IL-13 that activate Janus kinase (JAK)/signal transducer and activator of transcription (STAT) signaling leading to activation of STAT6 (26). STAT6 activation in HRS cells further increases TARC secretion (27). Together, this leads to a feedback loop of constant stimulation of HRS cells. In addition to its role in the pathogenesis of cHL, TARC is important as blood biomarker in both children and adults with cHL (11, 19, 28). Due to these reasons, it is worthwhile to further investigate the prognostic impact of TARC expression in a larger sample size.

We found an association between PD-L1 expression in the TME and interim remission status, which is not reported in the literature before. The PD-1-PD-L1 pathway plays an important role in the pathogenesis of cHL (29, 30). HRS cells express PD-L1 (and PD-L2) in part as a result of copy gain of chromosome 9p24.1 (29). HRS cells escape immune detection by over-expressing of PD-L1/PD-L2 (29, 30). Once engaged with PD-1 it suppresses T-cell effector function which contributes to immune evasion. PD-L1 expression is not only seen on HRS cells but also on tumor-associated macrophages (TAMs) (31). A recent study showed that TAMs in the proximity to HRS cells express high levels of PD-L1, likely in response to local cytokine production, and thereby significantly increase the total amount of PD-L1 in the vicinity of the malignant cells. Both on TAMs’ and HRS cells’ PD-L1 is available to bind PD-1 on CD4^+^ T cells and CD8^+^ T cells which augments the immunosuppression (31). This may explain why patients with cHL respond so well to PD-1/PD-L1 checkpoint inhibition (30, 32, 33). Our findings imply that PD-L1 expression specifically in the TME in pediatric cHL is correlated with outcome. Low expression of PD-L1 in the TME causing less immunosuppression and probably more vulnerability to treatment. However, as the lower of the CI is close to 0.5, interpretation is limited and therefore, this should be further investigated in a larger cohort of pediatric patients.

In our study, PD-L1 and PAX5 expression on HRS cells showed low discriminative power for DFS. Their role in pediatric patients is not clear yet, and this finding needs further assessment in a larger cohort of patients.

Remarkably, there are some differences between our data and previously published findings in children with cHL (See Supplementary Table S5 for a summary of the findings of previous studies). Barros et al. found a significant association between the expression of CD163 and the progression-free survival (PFS) (8), however, our study and the study of Gupta et al. found no significant association (10). The study of Barros had a lower number of patients with events (22% versus 33% in our study and 36% in the study of Gupta) (8, 10). Patients were treated according to other treatment protocols. Furthermore, a different clone of CD163 and a different scoring method was used in our study; expression patterns in our study were based on larger areas and exact percentages were calculated. Finally, we studied DFS instead of PFS. Gupta et al. found a significant association between CD30 expression and event-free survival (EFS) (9, 10). We found no association for CD30 with DFS. Gupta et al. had a comparable number of patients with an event (10). However, treatment protocols were different and not specified and a different scoring method was used. Due to the above-described differences between the studies, it is difficult to compare these studies. Strengths of our study were the large number of events and the scoring method based on larger areas and exact percentages that were calculated.

Interestingly, our results are in contrast with previous findings in adult patients. They mostly reported an adverse association between OS and EFS and the expression of PAX5 on the HRS cells, and CD68, CD163, PD-1, and PD-L1expression in the TME (4–7). However, differences between pediatric and adult studies have been described in previous studies (9, 10). These differences between pediatric cHL and adult cHL may be due to differences in pathogenesis, resulting from differences in the composition of the TME (12, 14–17). Barros et al. recently showed that the composition of the tumor environment in cHL differs between these two populations, with B lymphocytes outnumbering CD4 positive T lymphocytes in children but not in adults (14). Other studies found older age to be associated with higher percentages of CD68 and CD163 positive macrophages (34, 35). Moreover, differences may be due to the use of different treatment protocols in adult patients and due to the use of different antibodies and/or scoring methods.

We found high expression of CD30 and PD-L1 on HRS cells, and expression of PD-1 and PD-L1 in the TME in almost all patients. CD30, PD-L1 and PD-1 are all markers that can be targeted therapeutically (e.g., by brentuximab vedotin (BV) binding to CD30) (36, 37). Recently, a new treatment protocol with nivolumab (PD-1 inhibitor) and BV was implemented in a large part of the United States and Europe for pediatric patients with cHL with failure of first line therapy (NCT02927769). Furthermore, Pembrolizumab, a PD-1 inhibitor, was added to first-line treatment in pediatric patients with advanced disease (NCT03407144). The expression in pediatric patients underscores the need for further investigations of these novel therapies in first line treatment in children.

Our study is the first to examine eight different markers simultaneously. Another strength of our data is the event-enriched setting particularly in pediatric cHL patients. However, due to the low relapse rate in cHL, we included patients diagnosed over a period of 18 years and therefore treated with different treatment regimens. Despite this event-enriched cohort, the study still lacked the statistical power to perform a sub-analysis per treatment protocol for the primary outcome. Another possible limitation of the study is the use of immunohistochemical staining. This can lead to inconsistent results depending on differences in tissue fixation, the type of antibody and staining method, the scoring method, and the observer’s interpretation. To minimize these inconsistencies, we based our scoring method on published previous studies (4, 7, 13, 17) and we tried to overcome the interobserver variability by using a dual-head microscope and reviewing tissue samples together.

In conclusion, we found that low CD15 and TARC expression on HRS cells are associated with treatment failure. Furthermore, we found that PD-L1 expression in the TME at diagnosis is associated with CR status at interim analysis. Both these outcomes should be further investigated. Furthermore, our data demonstrate differences in the expression patterns and prognostic impact of immunohistochemical markers between pediatric and adult patients with cHL. Further research into these differences may lead to specific prognostic factors in pediatric cHL, indispensable for improvement of treatment in this population.

## Data Availability

The raw data supporting the conclusion of this article will be made available by the authors, without undue reservation.

## Abbreviations

AUC, area under the curve; cHL, classical Hodgkin lymphoma; CR, complete remission; CT, computed tomography; DFS, disease-free survival; EBV, Epstein Barr virus; EFS, event-free survival; HRS, Hodgkin and Reed-Sternberg; IL, interleukin; JAK/STAT, Janus Kinase/signal transducer and activator of transcription; OR, odds ratio; PAX-5, paired box 5; PD-1, programmed death 1; PD-L1, programmed death ligand 1; PET, positron emission tomography; PFS, progression free survival; PHL, pediatric Hodgkin lymphoma; ROC, receiver operating curve; TARC, thymus and activation-regulated chemokine; Th2, T Helper 2 cell; TME, tumor microenvironment.
