# Supplementary material for: The Prognostic Value of Eight Immunohistochemical Markers Expressed in the Tumor Microenvironment and on Hodgkin Reed-Sternberg Cells in Pediatric Patients With Classical Hodgkin Lymphoma
Source: Pathol Oncol Res. 2022 Aug 11;28:1610482. doi: 10.3389/pore.2022.1610482 (PMC9402887; doi:10.3389/pore.2022.1610482)
Supplement: Supplementary file 1 [file DataSheet1.docx]

**Supplemental tables**

**SUPPLEMENTAL TABLE 1. Immunohistochemical staining procedures for the different immunohistochemical markers**

| Antibody | **Type** | **Concentration** | **Company** | **Clone** | **Lot number** | **Procedure** | **Ab incubation time** |
| --- | --- | --- | --- | --- | --- | --- | --- |
| CD15 | Anti-Mouse | 11 µg/ml | Ventana | *MMA* | G29761 | Ultraview CC1 64' | 32 minutes |
| CD30 | Anti-Mouse | 1.23 µg/ml | Ventana | BerH2 | H03152 | Optiview CC1 80' | 90 minutes |
| CD68 | Anti-Mouse | 0.4 µg/ml | Ventana | KP-1 | 423029 | Optiview CC1 16' | 32 minutes |
| CD163 | Anti-Mouse | 0.50 µg/ml | Cell Marque | MRQ-26 | V0002105 | Optiview CC1 32' | 12 minutes |
| PD-1 | Anti-Mouse | 3.37 µg/ml | Cell Marque | NAT105 | 1619003B | Optiview CC1 16' | 12 minutes |
| PD-L1 | Anti-Rabbit | 1.61 µg/ml | Ventana | SP263 | G17141 | Optiview CC1 64' | 32 minutes |
| TARC | Anti-Goat | 0.2 mg/ml | R&D systems | Hccl17 | AR40317631 | Ultraview CC1 52' | 32 minutes |
| PAX5 | Anti-Mouse | 0.4 µg/ml | DAKO | M7307 | 20061060 | Optiview CC1 64' | 52 minutes |

Abbrevations: PD-1 Programmed Death 1; PD-L1 Programmed Death Ligand 1; TARC Thymus and Activation-Regulated Chemokine; PAX5 Paired Box 5; µg microgram; mg milligram; ml millilitre; MMA Mouse Monoclonal Antibody; CC1 Cell Conditioning solution 1.

**SUPPLEMENTAL TABLE 2. Baseline characteristics for the whole cohort (left), the primary outcome analysis (middle), and the secondary outcome analysis (right)**

|  | | | | | | | | |
| --- | --- | --- | --- | --- | --- | --- | --- | --- |
|  | Total  (n=67) | Treatment success  (n=45) | Treatment failure  (n=22) | p-value | Complete remission  (n=37) | No complete remission  (n=30) | p-value |  |
| Sex, n **(**%) |  |  |  | 0.141 ₸ |  |  | 0.012₸ |  |
| Male | 31 (46.3) | 18 (40.0) | 13 (59.1) |  | 12 (32.4) | 19 (63.3) |  |  |
| Female | 36 (53.7) | 27 (60.0) | 9 (40.9) |  | 25 (67.6) | 11 (36.7) |  |  |
| Age, mean (SD) |  |  |  | 0.095 † |  |  | 0.212 Ῡ |  |
|  | 13.64  (± 3.19) | 13.09  (± 3.56) | 14.76  (± 1.84) |  | 14.08  (± 2.79) | 13.10  (± 3.59) |  |  |
| Histologic subtype, n **(**%) |  |  |  | 0.350 * |  |  | 0.649 * |  |
| Nodular sclerosis | 36 (87.8) | 18 (81.8) | 18 (94.7) |  | 20 (90.9) | 16 (84.2) |  |  |
| Other | 5 (12.2) | 4 (18.1) | 1 (5.3) |  | 2 (9.1) | 3 (15.8) |  |  |
| Not classifiable**/**  unknown | 26 | 23 | 3 |  | 15 | 11 |  |  |
| Stage, n **(**%) |  |  |  | 0.627 ₸ |  |  | 0.033 ₸ |  |
| IA - IIB | 38 (57.6) | 25 (55.6) | 13 (61.9) |  | 25 (69.4) | 13 (43.3) |  |  |
| IIIA - IVB | 28 (42.4) | 20 (44.4) | 8 (38.1) |  | 11 (30.6) | 17 (56.7) |  |  |
| Missing | 1 | 0 | 1 |  | 1 | 0 |  |  |
| Treatment protocol n (%) |  |  |  | <0.001 ₸ |  |  | 0.007 ₸ |  |
| EuroNet-PHL-  C1/C2 | 45 (67.2) | 38 (84.4) | 7 (31.8) |  | 30 (81.1) | 15 (50.0) |  |  |
| Other^1^ | 22 (32.8) | 7 (15.6) | 15 (68.2) |  | 7 (18.9) | 15 (50.0) |  |  |
| Complete remission at interim PET scan, n (%) |  |  |  | 0.607 ₸ |  |  | NA |  |
| Yes | 37 (55.2) | 26 (57.8) | 11 (50.0) |  | NA | NA |  |  |
| No | 30 (44.8) | 19 (42.2) | 11 (50.0) |  | NA | NA |  |  |
| Radiation therapy, n (%) |  |  |  | 0.017 ₸ |  |  | 0.007 ₸ |  |
| Yes | 26 (38.8) | 13 (28.9) | 13 (59.1) |  | 9 (24.3) | 17 (56.7) |  |  |
| No | 41 (61.2) | 32 (71.1) | 9 (59.1) |  | 28 (75.7) | 13 (43.3) |  |  |

Abbreviations: n number of counts; SD standard deviation; NA not applicable; EuroNet-PHL European Network-Paediatric Hodgkin Lymphoma.

^1^ Other treatment protocols or treatment regimens that were used were the Children’s Oncology Group (COG) protocol, the HD84 Sophia protocol, the Stichting Nederlandse Werkgroep Leukemie Bij Kinderen-Anaplastic Large Cell Lymphoma (ALCL99) protocol, and the ABVD-regimen (adriamycin, bleomycin, vinblastine, and dacarbazine)

₸ For these variables the Pearson Chi-Square test was used.

* For these variables the Fisher’s exact test was used since >20% of the expected count was <5 and/or the minimum expected count was <1.

Ῡ For these variables the Independent Student’s t-test was used.
† For these variables the Mann-Whitney test was used because the assumptions for Independent Student's t-test were not met.

**SUPPLEMENTAL TABLE 3. Expression patterns of the different biomarkers regarding patients who experienced early relapse versus patients who did not experience early relapse**

|  | | | | | |
| --- | --- | --- | --- | --- | --- |
|  | **Early relapse (< 1 year)**  **Value n*** | | **Late relapse (> 1 year)**  **Value n*** | | **P Value** |
| Markers expressed on HRS cells (% positive cells) |  |  |  |  |  |
| PAX5  Median (IQR) | 83.00  (71.00 – 92.00) | 10 | 71.00  (47.50 – 83.00) | 12 | 0.074 |
| CD15  Median (IQR) | 51.00  (31.50 – 61.00) | 10 | 56.00  (17.50 – 88.00) | 12 | 0.551 |
| CD30  Median (IQR) | 91.00  (87.00 – 96.50) | 10 | 96.00  (87.00 – 96.00) | 12 | 0.663 |
| PD-L1  Median (IQR) | 97.00  (90.50 – 98.50) | 10 | 94.00  (75.00 – 96.00) | 12 | 0.076 |
| TARC  Median (IQR) | 90.00  (81.50 – 94.00) | 10 | 88.00  (1.50 – 91.50) | 12 | 0.174 |
| Markers in TME  (score 0-4) |  |  |  |  |  |
| CD163  Mean (SD) | 2.26 (1.34) | 10 | 2.53 (0.77) | 12 | 0.691 |
| CD68  Mean (SD) | 2.28 (0.75) | 10 | 2.45 (0.98) | 12 | 0.921 |
| PD-1  Mean (SD) | 1.24 (1.00) | 10 | 1.18 (1.01) | 12 | 0.791 |
| PD-L1  Mean (SD) | 2.44 (0.80) | 10 | 2.18 (1.08) | 12 | 0.620 |

Abbreviations: n number of counts; HRS Hodgkin and Reed-Sternberg; TME tumour microenvironment; IQR interquartile range; SD standard deviation; PAX5 Paired Box 5; PD-L1 Programmed Death Ligand 1; TARC Thymus and Activation-Regulated Chemokine; PD-1 Programmed Death 1.

**SUPPLEMENTAL TABLE 4. Expression patterns of the different immunohistochemical markers regarding achievement of complete remission the interim PET scan**

|  | | | | | |
| --- | --- | --- | --- | --- | --- |
|  | **Complete remission at the interim PET scan**  **Value n*** | | **No complete remission at the interim PET scan**  **Value n*** | | **P Value** |
| Markers expressed on HRS cells (% positive cells) |  |  |  |  |  |
| PAX5  Median (IQR) | 82.00  (71.00 - 94.00) | 37 | 84.00  (46.00 - 92.00) | 30 | 0.216 * |
| CD15  Median (IQR) | 86.00  (45.50 - 96.00) | 36 | 62.00  (39.00 - 89.50) | 30 | 0.152 * |
| CD30  Median (IQR) | 94.00  (88.00 - 97.00) | 37 | 95.00  (87.50 - 96.00) | 30 | 0.157 † |
| PD-L1  Median (IQR) | 96.00  (92.00 – 100.00) | 37 | 96.00  (93.50 - 98.00) | 30 | 0.807 * |
| TARC  Median (IQR) | 92.00  (88.00 - 94.00) | 37 | 89.00  (77.50 - 94.00) | 30 | 0.175 * |
| Markers in TME  (score 0-4) |  |  |  |  |  |
| CD163  Mean (SD) | 2.12 (1.08) | 37 | 2.13 (0.93) | 30 | 0.993 * |
| CD68  Mean (SD) | 2.43 (0.73) | 37 | 2.41 (0.71) | 30 | 0.915 * |
| PD-1  Mean (SD) | 1.32 (0.90) | 37 | 1.59 (1.02) | 30 | 0.259 * |
| PD-L1  Mean (SD) | 2.43 (0.69) | 37 | 2.71 (0.95) | 30 | 0.165 * |

Abbreviations: PET Positron Emission Tomography; n number of counts; HRS Hodgkin and Reed-Sternberg; IQR interquartile range; SD standard deviation; PAX5 Paired Box 5; PD-L1 Programmed Death Ligand 1; TARC Thymus and Activation-Regulated Chemokine; PD-1 Programmed Death 1.

* For these variables the Independent Student’s t-test was used.

† For this variable the Welch’s t-test was used because the assumptions for Independent Student's t-test were not met.

**SUPPLEMENTAL TABLE 5. Characteristics of previous studies performed in a pediatric population**

| Study, year | **Country** | **Number of subjects (events, n (%))** | **Median age (years)** | **Stage, n (%)** | **Treatment** | **Antibodies (clone)** | **Scoring** | **Threshold(s)** | **Univariate prognostic impact** | **Multivariable prognostic impact** |
| --- | --- | --- | --- | --- | --- | --- | --- | --- | --- | --- |
| Dinand et al., 2008 (1) | India | 121 (CR 112 (92.6%); PR 3 (2.5%); progression 2 (1.6%); early death 4 (3.3%)) | 8 | Stage I/IIa 35 (29%);  Stage IIb/IV 86 (71%) | COPP, COPP/ABVD | CD15, CD20, CD30, CD45, CD45RO (all DakoCytomation, NOS) | Visual, counting | NOS | CD15 positive (PFS and OS); CD20, NSS; CD30, NSS; CD45, NSS; CD45RO, NSS | CD15 positive (PFS and OS); CD20, NSS; CD30, NSS; CD45, NSS; CD45RO, NSS |
| Barros et al., 2012 (2) | Brazil | 95 (PFS at 60 months was 78.6%) | 14 | Stage I/II 59 (62.1%);  Stage III/IV 36 (37.9%) | ABVD-protocol, HD90 protocol | CD14 (clone 7); CD68 (PGM1); CD163 (10D6); CD83 (1H4b); CD207 (12D6) | Computer-assisted microscopic analysis | 25th and 50th percentiles | CD14, NSS; CD68, NSS; CD163, adverse (PFS); CD83, NSS;  CD207, NSS | CD14, NSS; CD68, NSS; CD163, NSS; CD83, NSS; CD207, NSS |
| Gupta et al., 2013 (3) | Canada | 96 (progressive disease, relapse or death 35 (36.5%)) | 14 | Stage I/II 50 (52.1%);  Stage III/IV 45 (46.9%) | Various regimes, NOS | CD30 (BerH2); CD68 (KP1); CD163 (10D6) | Visual, estimation | ≥5% positive cells relative to overall cellularity | CD30, adverse (EFS); CD68, NSS; CD163, NSS | CD30, NSS; CD68, NSS; CD163, NSS |
| Jimenez et al., 2021 (4) | Argentina | 80 (no number of events mentioned) | 9.5 | Unknown | Unknown | PD-1:CD279 (NAT); PD-L1:CD274 (4E54) | Computer-assisted | 50th percentile | PD-1, NSS; PD-L1, NSS | PD-1, NSS; PD-L1, NSS |

Abbreviations: N number of counts; CR complete response; PR partial response; COPP cyclophosphamide, vincristine, procarbazine, prednisolone; ABVD-protocol consists of adriamycin, bleomycin, vinblastine, dacarbazine; HD90-protocol consists of OPPA (vincristine, procarbazine, prednisone and doxorubicine) for girls, OEPA (vincristine, etoposide, prednisone and doxorubicine) for boys, and COPP (cyclophosphamide, vincristine, procarbazine and prednisone) for both when not presenting with early stages disease; NOS Not Otherwise Specified; NSS Not Statically Significant.

**Supplemental figures**


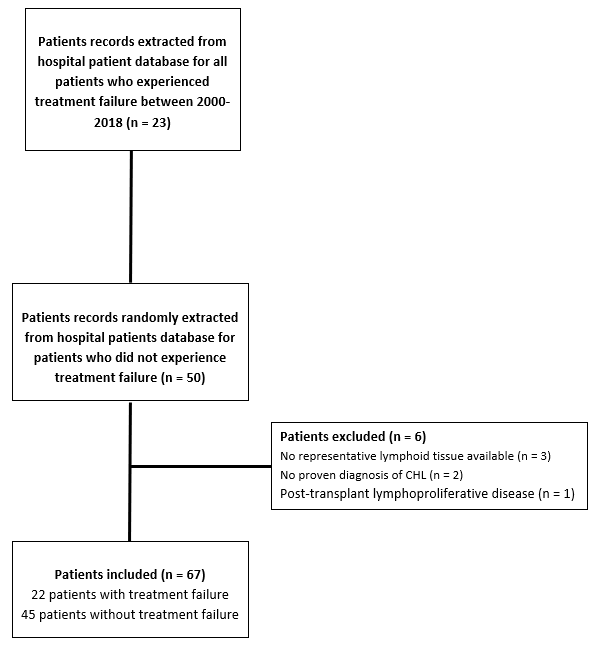


**Supplemental Figure 1. Flowchart of patient selection.** We extracted patients records from the hospital patients database for 73 patients. 67 patients (91.8%) met the inclusion criteria. Six patients were excluded from the study; three patients had no representative lymphoid tissue available, two patients had no proven diagnosis of cHL, and one patient was excluded because of post-transplant lymphoproliferative disease (PTLD).

**
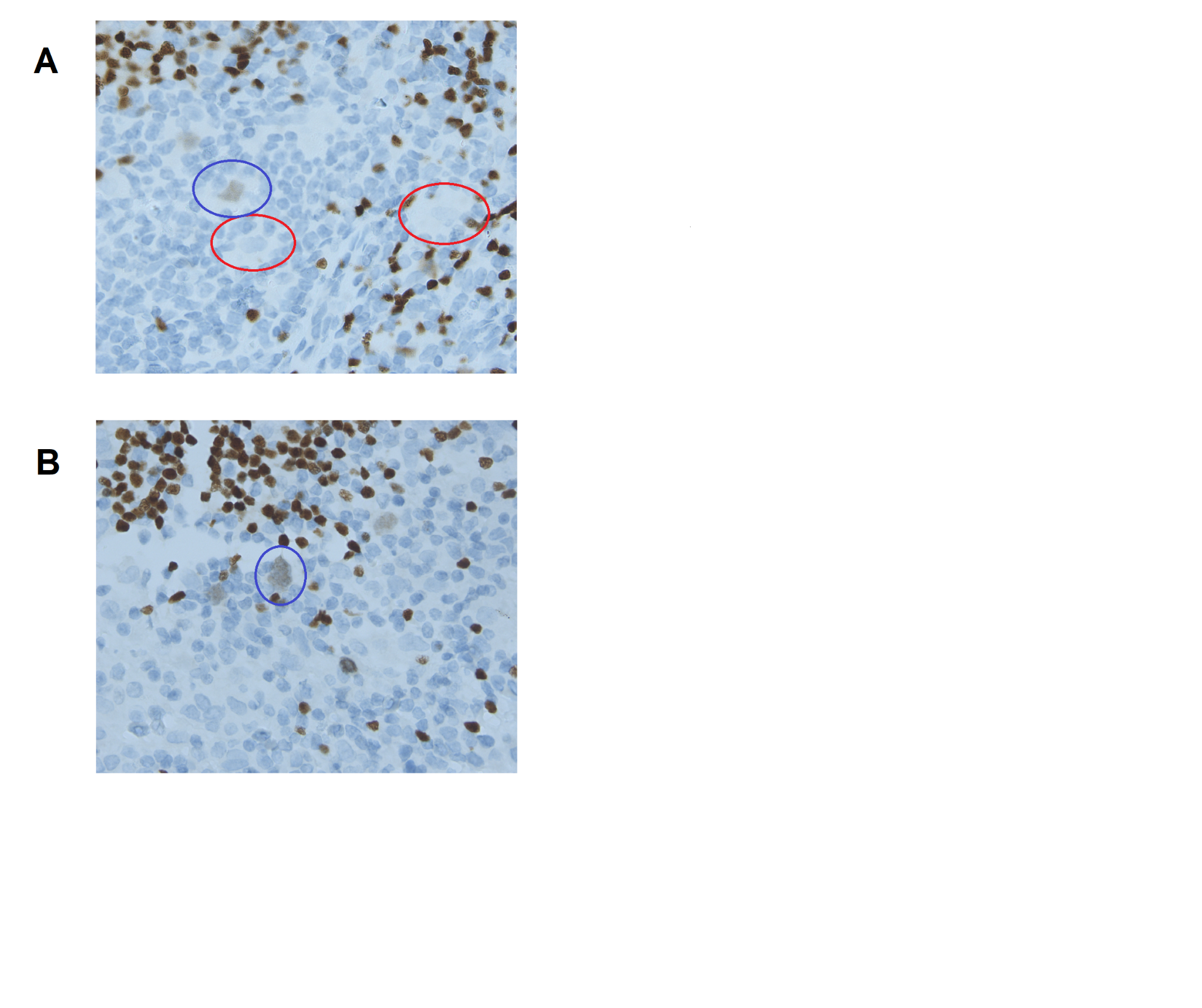
**

**Supplemental Figure 2. Immunohistochemical analysis of PAX5 expression by HRS cells.**

2A. and 2B. HRS cells were stained for PAX5. The HRS cells circled in blue were counted as positive, in red as negative. All pictures were taken with a x40 objective.


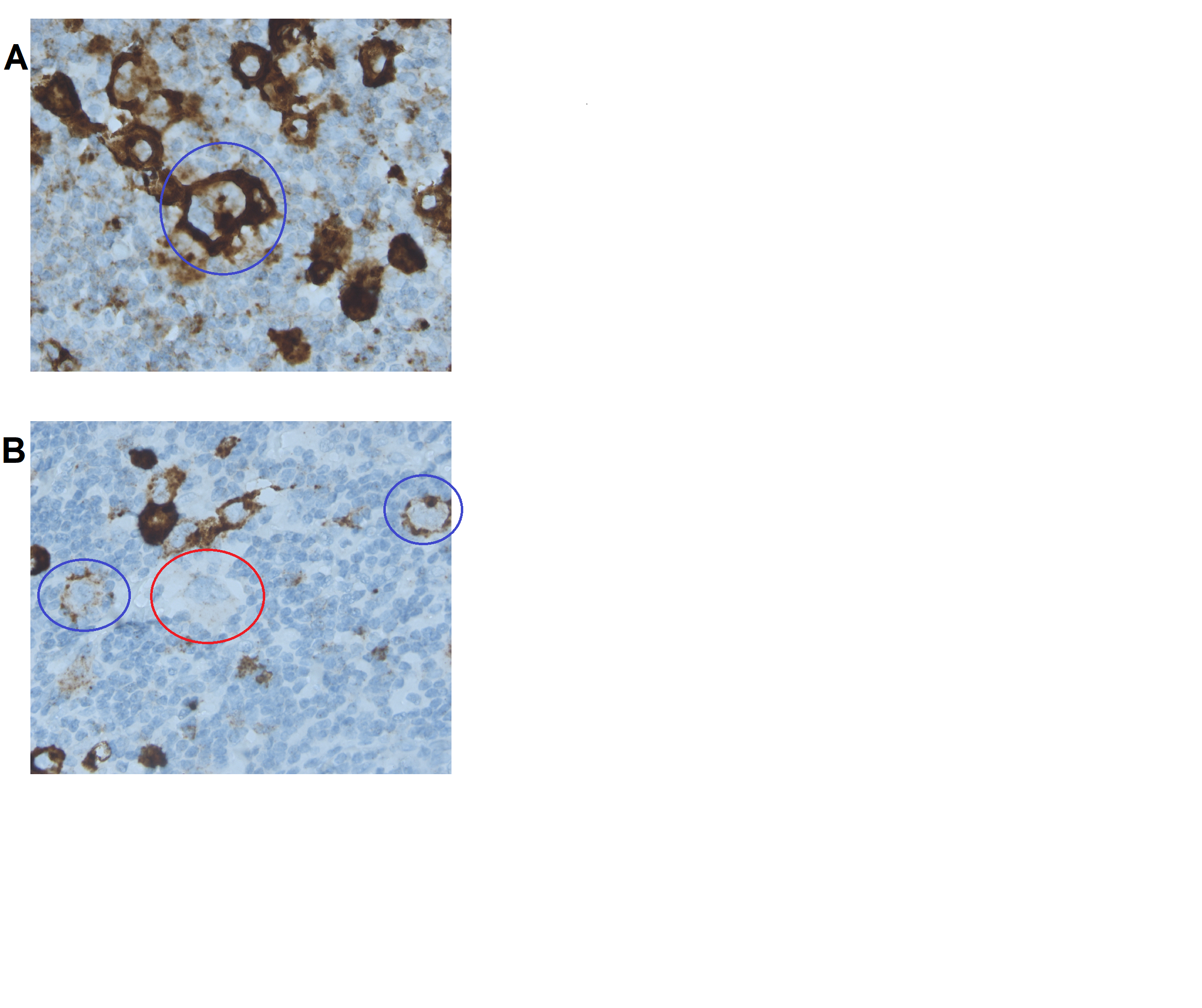


**Supplemental Figure 3. Immunohistochemical analysis of CD30 expression by HRS cells.** 3A and 3B. HRS cells were stained for CD30. The HRS cells circled in blue were counted as positive, in red as negative. All pictures were taken with a x40 objective.


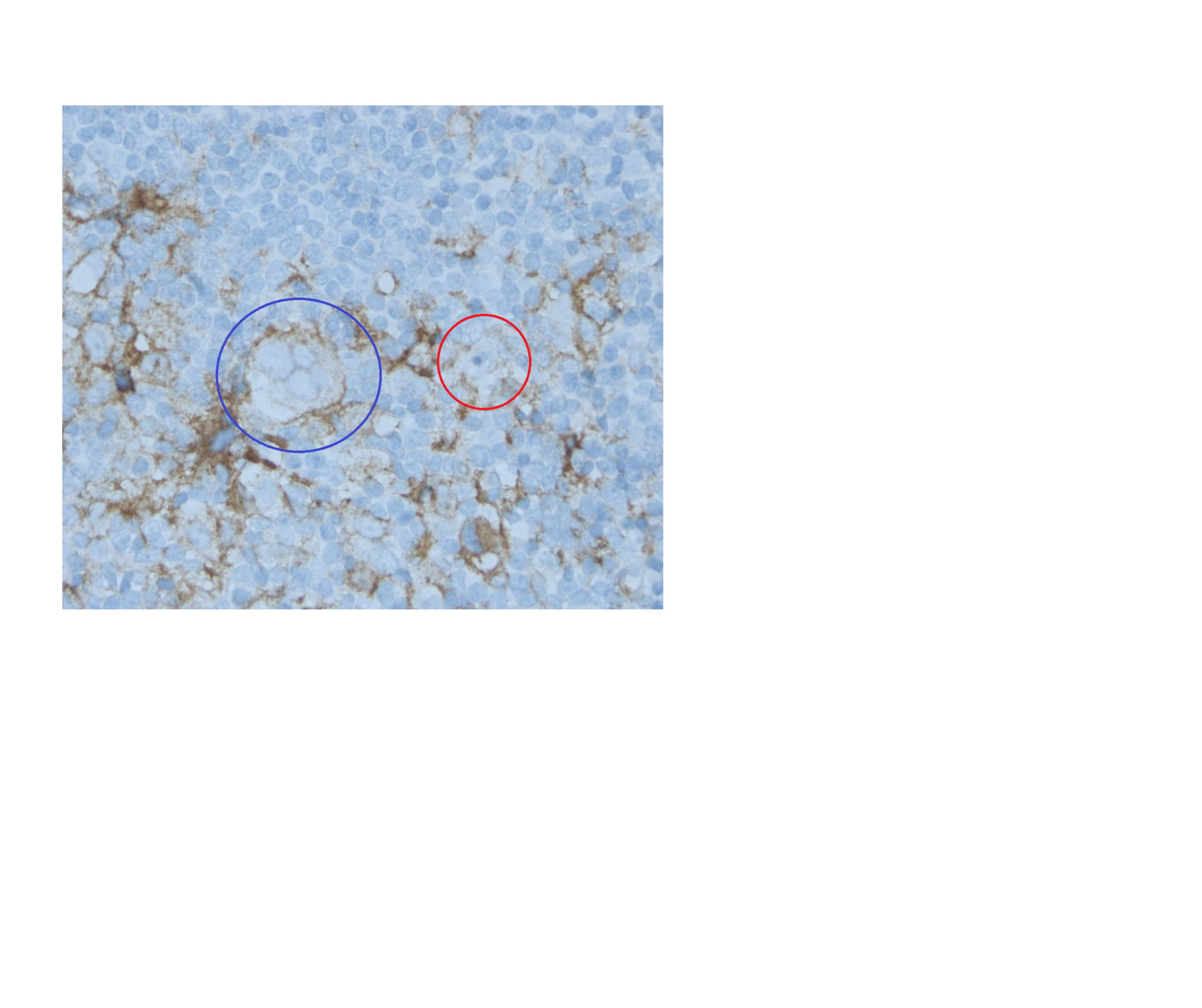


**Supplemental Figure 4. Immunohistochemical analysis of PD-L1 expression by HRS cells.**

HRS cells were stained for PD-L1. The HRS cell circled in blue was counted as positive. The HRS cell circled in red is counted as negative, since it showed no continuous positive membrane staining. The focal patchy positivity is due to positivity of the adjacent immunoreactive cells. All pictures were taken with a x40 objective.


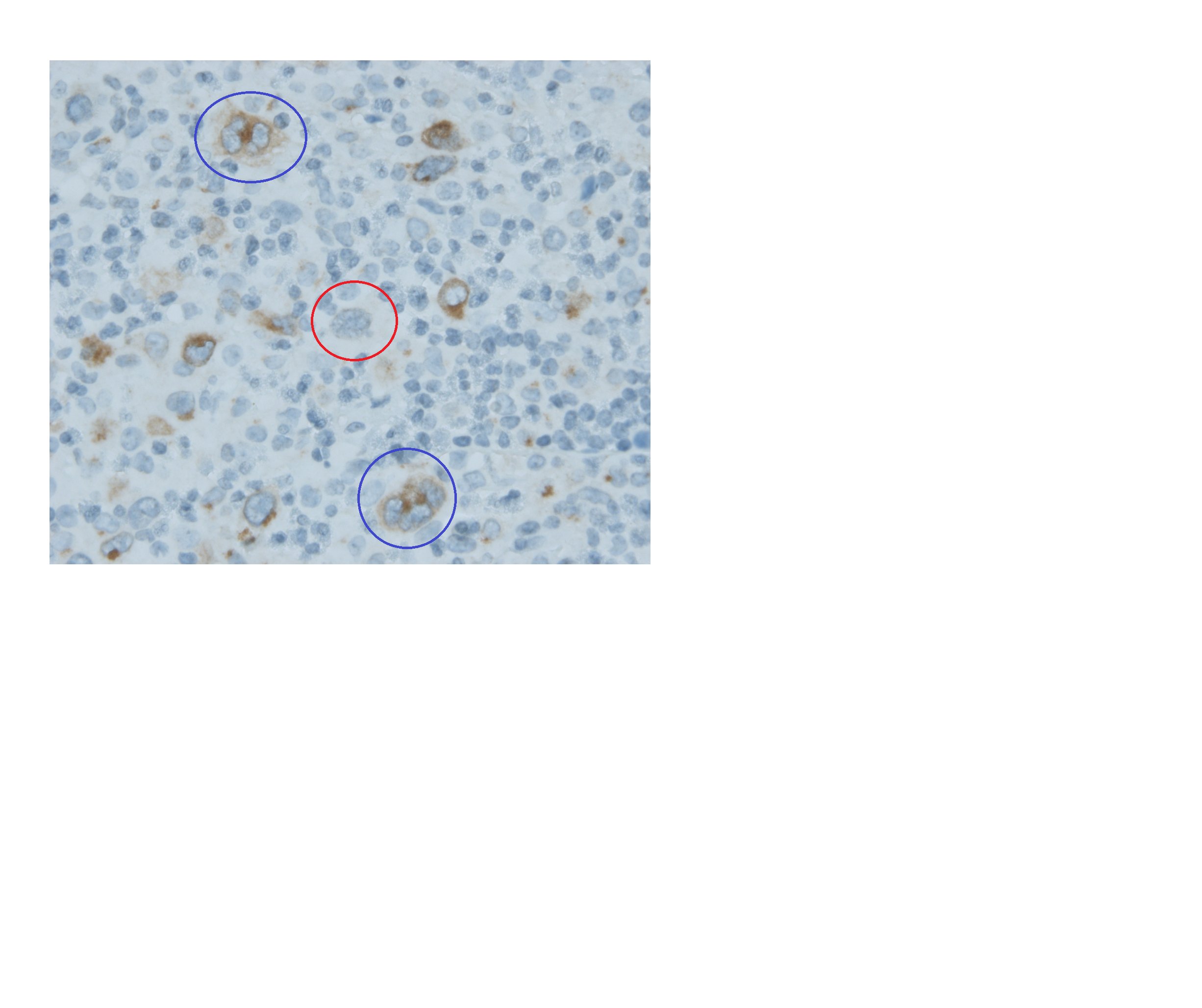


**Supplemental Figure 5. Immunohistochemical analysis of TARC expression by HRS cells.**

HRS cells were stained for TARC. The HRS cell circled in blue were counted as positive, in red as negative. All pictures were taken with a x40 objective.


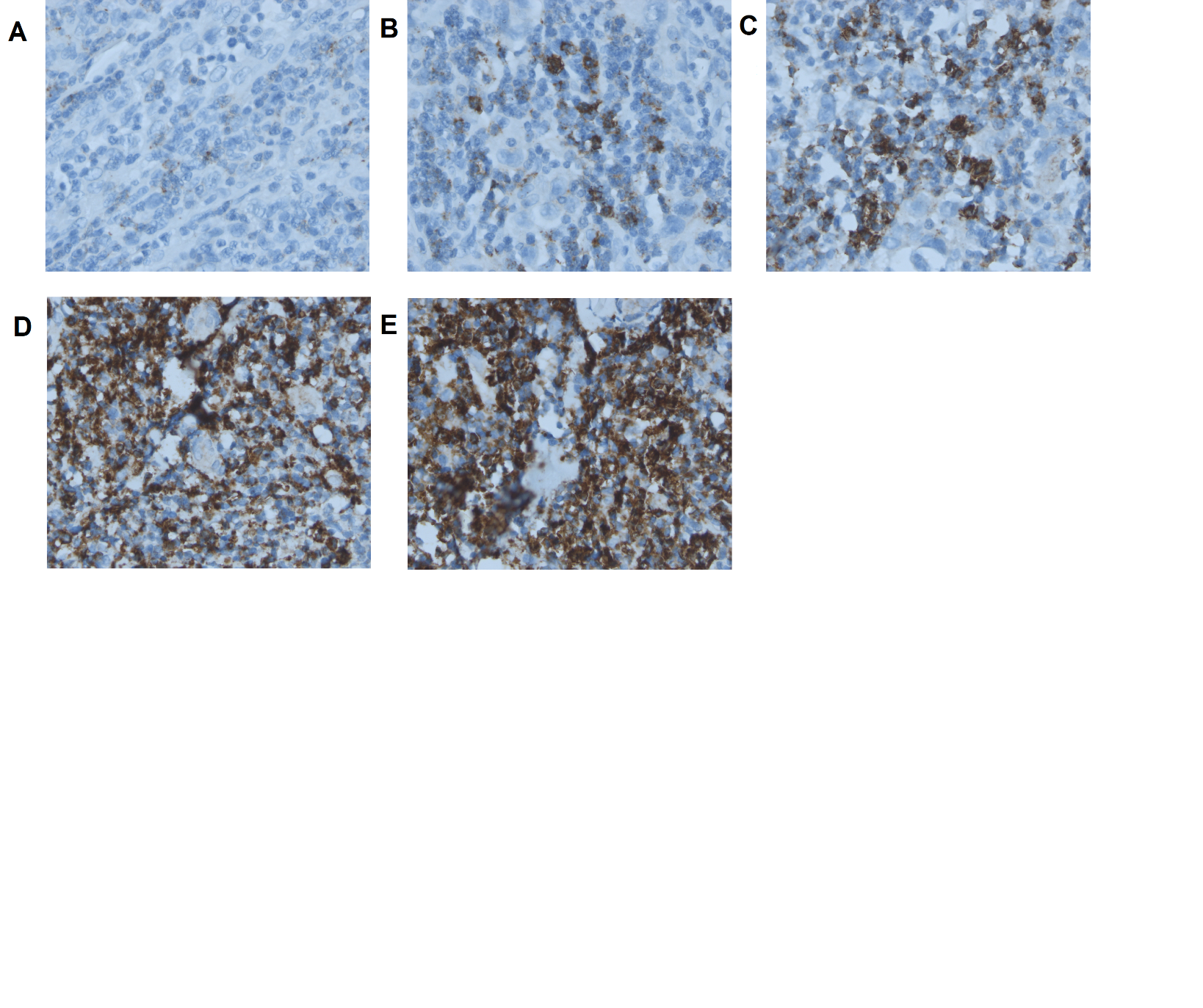


**Supplemental Figure 6. Immunohistochemical analysis of PD-1 expressed in the tumour microenvironment.** This figure shows the estimates of the positive staining cells for PD-1 in relation to the overall cellularity according to the five groups: 0-5% score 0 (A), 6-25% score 1 (B), 26-50% score 2 (C), 51-75% score 3 (D), and >75% score 4 (E). All pictures were taken under a x40 objective.


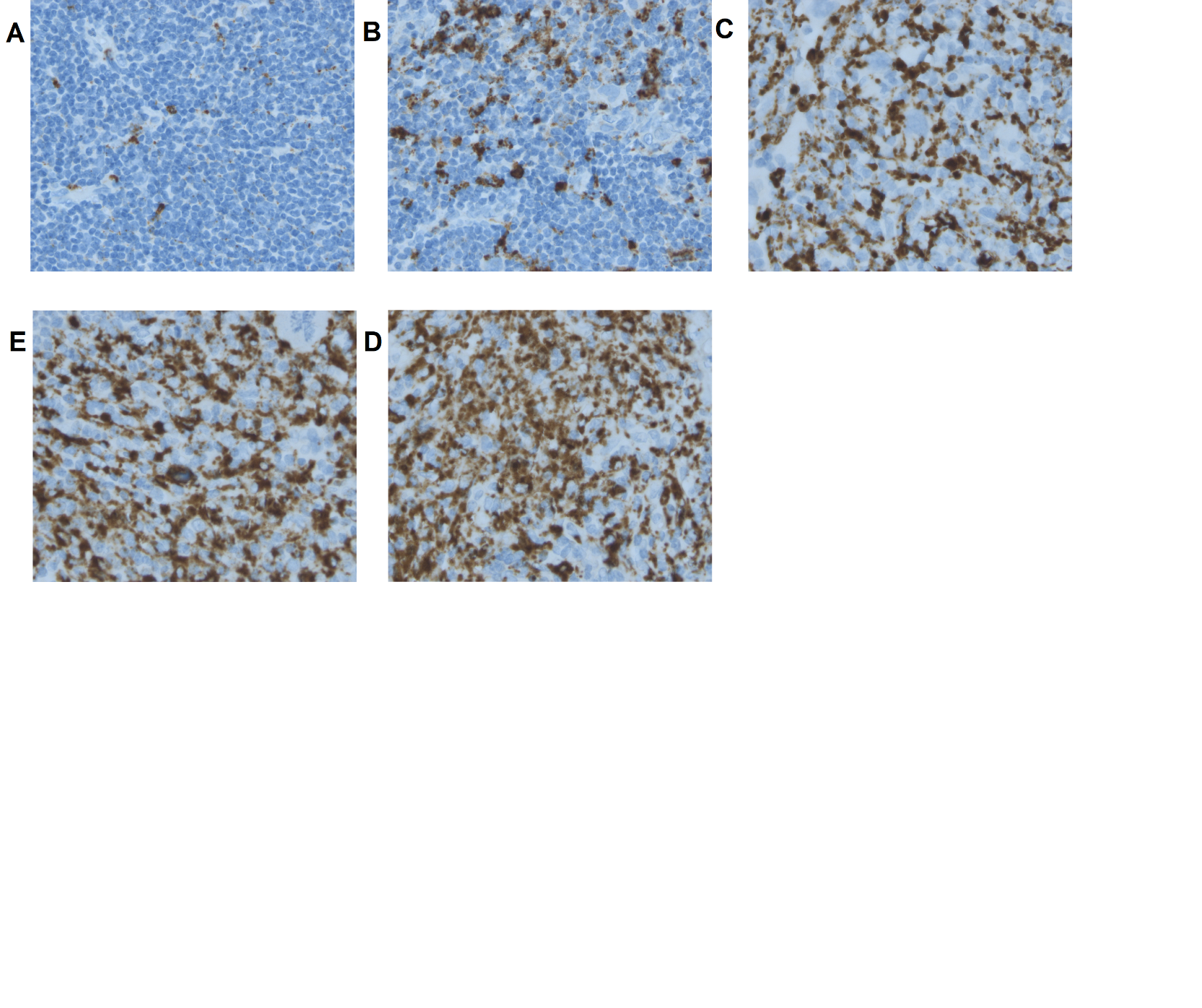


**Supplemental Figure 7. Immunohistochemical analysis of CD68 expressed in the tumour microenvironment.** This figure shows the estimates of the positive staining cells for CD68 in relation to the overall cellularity according to the five groups: 0-5% score 0 (A), 6-25% score 1 (B), 26-50% score 2 (C), 51-75% score 3 (D), and >75% score 4 (E). All pictures were taken under a x40 objective.


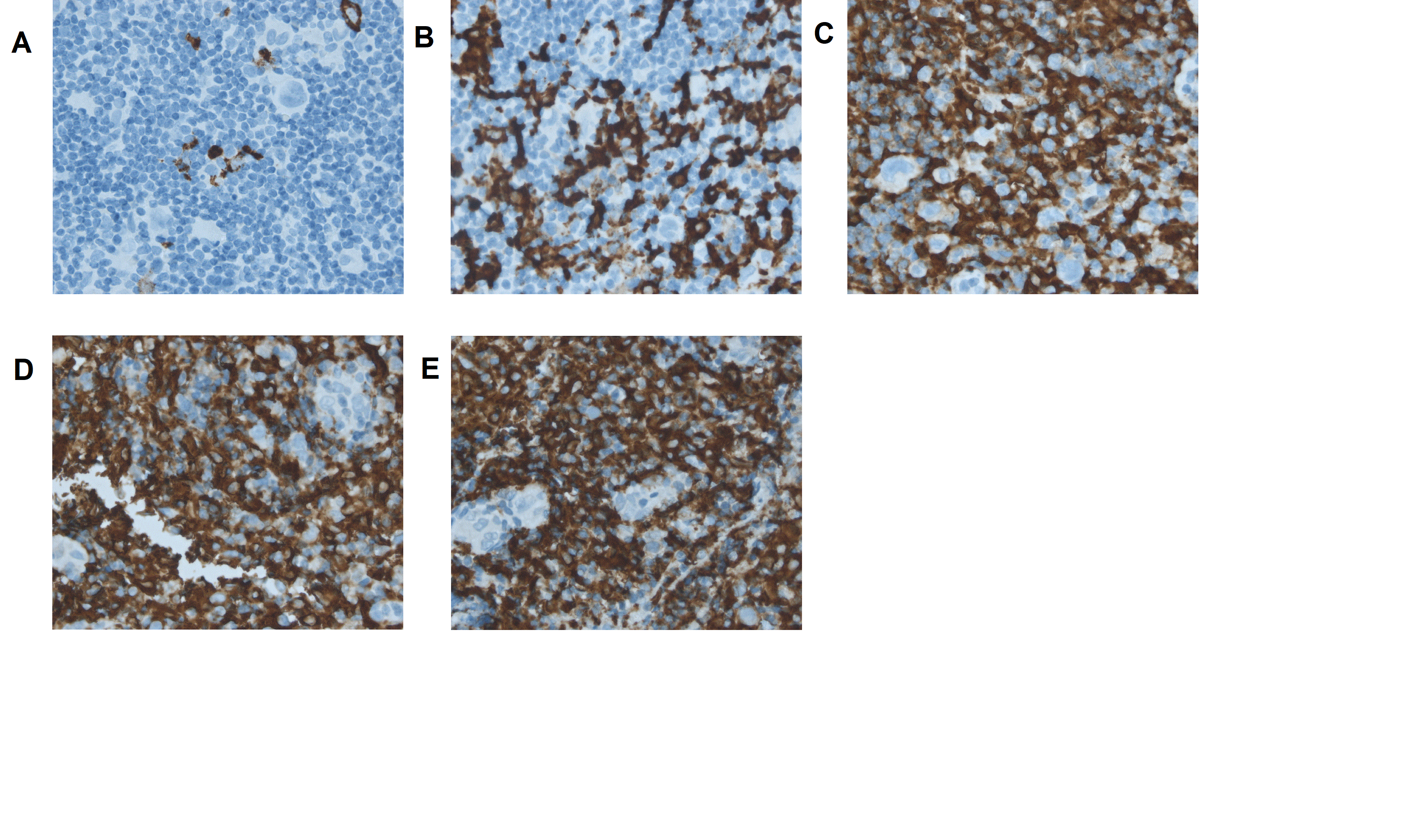


**Supplemental** **Figure 8. Immunohistochemical analysis of CD163 expressed in the tumour microenvironment.** This figure shows the estimates of the positive staining cells for CD163 in relation to the overall cellularity according to the five groups: 0-5% score 0 (A), 6-25% score 1 (B), 26-50% score 2 (C), 51-75% score 3 (D), and >75% score 4 (E). All pictures were taken under a x40 objective.


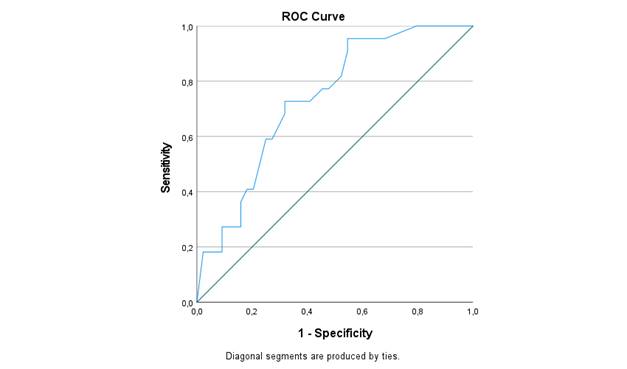


1. **CD15**


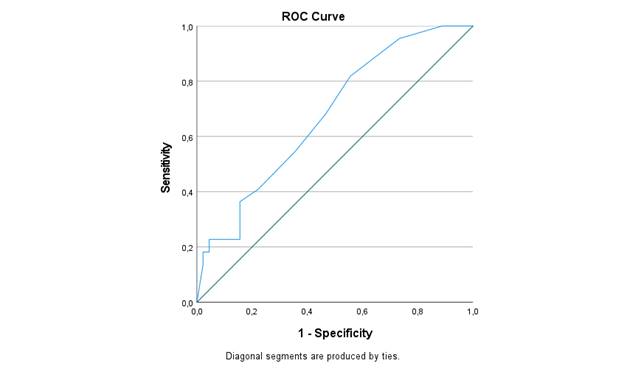


1. **TARC**

**Supplemental Figure 9. Evaluation of the discriminating power of CD15 and TARC expression for DFS by ROC curves.** A. CD15 showed adequate discriminative power for DFS, with an AUC of 0.74, 95% CI 0.62-0.86). B. TARC also showed lower discriminative power for DFS, with an AUC of 0.68, 95% CI 0.54-0.81.


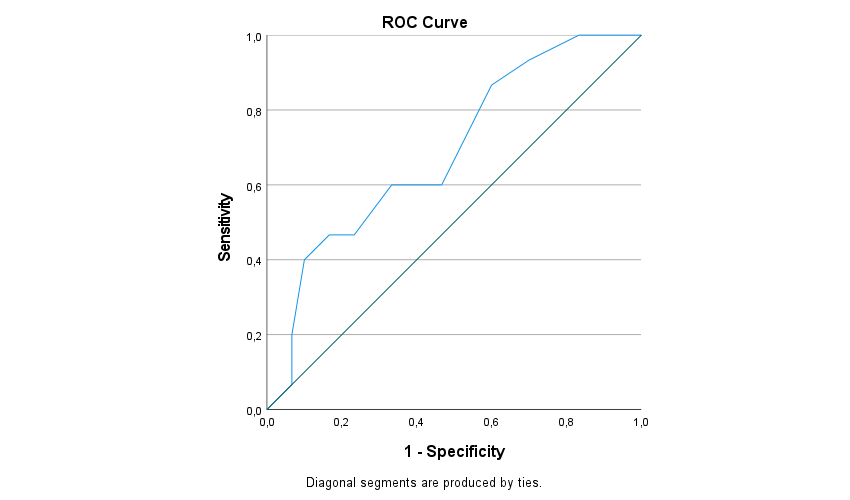


**Supplemental Figure 10. Evaluation of the discriminating power of PD-L1 expression in the TME for interim remission status by ROC curve.** PD-L1 showed discriminative power for interim remission status, with an AUC of 0.69 (95% CI 0.53-0.85)

**References**

1. Dinand V, Malik A, Unni R, Arya LS, Pandey RM, Dawar R. Proliferative index and CD15 expression in pediatric classical Hodgkin lymphoma. Pediatr Blood Cancer. 2008;50(2):280-3.

2. Barros MH, Hassan R, Niedobitek G. Tumor-associated macrophages in pediatric classical Hodgkin lymphoma: association with Epstein-Barr virus, lymphocyte subsets, and prognostic impact. Clin Cancer Res. 2012;18(14):3762-71.

3. Gupta S, Yeh S, Chami R, Punnett A, Chung C. The prognostic impact of tumour-associated macrophages and Reed-Sternberg cells in paediatric Hodgkin lymphoma. Eur J Cancer. 2013;49(15):3255-61.

4. Jimenez O, Colli S, Garcia Lombardi M, Preciado MV, De Matteo E, Chabay P. Epstein-Barr virus recruits PDL1-positive cells at the microenvironment in pediatric Hodgkin lymphoma. Cancer Immunol Immunother. 2021;70(6):1519-26.
